# Root-associated fungal community reflects host spatial co-occurrence patterns in a subtropical forest

**DOI:** 10.1038/s43705-021-00072-6

**Published:** 2021-11-06

**Authors:** Jialiang Kuang, Shun Han, Yongjian Chen, Colin T. Bates, Pandeng Wang, Wensheng Shu

**Affiliations:** 1 Institute for Environmental Genomics and Department of Microbiology and Plant Biology, University of Oklahoma, Norman, OK USA; 2 State Key Laboratory of Biocontrol, Guangdong Key Laboratory of Plant Resources and Conservation of Guangdong Higher Education Institutes, College of Ecology and Evolution, Sun Yat-sen University, Guangzhou, China; 3 Department of Environmental Science, University of Arizona, Tucson, AZ USA; 4 School of Life Sciences, South China Normal University, Guangzhou, China

**Keywords:** Community ecology, Microbial ecology, Fungal ecology, Forest ecology

## Abstract

Plant roots harbor and interact with diverse fungal species. By changing these belowground fungal communities, focal plants can affect the performance of surrounding individuals and the outcome of coexistence. Although highly host related, the roles of these root-associated fungal communities per se in host plant spatial co-occurrence is largely unknown. Here, we evaluated the host dependency of root-associated communities for 39-plant species spatially mapped throughout a 50-ha subtropical forest plot with relevant environmental properties. In addition, we explored whether the differentiation in root fungal associations among plant species can reflect their observed co-occurrence patterns. We demonstrated a strong host-dependency by discriminating the differentiation of root-associated fungal communities regardless of background soil heterogeneity. Furthermore, Random Forest modeling indicated that these nonrandom root fungal associations significantly increased our ability to explain spatial co-occurrence patterns, and to a greater degree than the relative abundance, phylogenetic relatedness, and functional traits of the host plants. Our results further suggested that plants harbor more abundant shared, “generalist” pathogens are likely segregated, while hosting more abundant unique, “specialist” ectomycorrhizal fungi might be an important strategy for promoting spatial aggregation, particularly between early established trees and the heterospecific adults. Together, we provide a conceptual and testable approach to integrate this host-dependent root fungal “fingerprinting” into the plant diversity patterns. We highlight that this approach is complementary to the classic cultivation-based scheme and can deepen our understanding of the community-level effect from overall fungi and its contribution to the pairwise plant dynamics in local species-rich communities.

## Introduction

A wide range of fungal species, including beneficial ones (e.g., mycorrhizal fungi) and plant pathogens, can influence the performance of their hosts, including survival, growth rate, reproduction, and competitiveness [1–3]. In addition, evidence is mounting that the diversity maintenance and population dynamics in plant communities are mediated by plant-soil feedbacks [4–13], whose direction and strength may depend on how plants structure and shape the belowground fungal communities and interact with them [3, 10, 11]. Previous studies of plant-fungi interaction have mainly focused on how plants alter belowground fungi throughout the root interface, the rhizosphere, and microbial feedbacks on plant dynamics [1, 5]. However, relatively little attention has been devoted to the roles of root-associated fungal communities per se in host plant spatial co-occurrence, despite the fact that diverse fungal species have been associated and coevolved with most plant species in nature and are host-specific [2, 3, 14–16].

These highly host-related fungi influence the plant’s growth and fitness [5]. They could be recruited in the surrounding soil by the focal hosts as they grow and cause plant-soil feedbacks when plants are growing together. Within the existing theoretical framework of plant-soil feedbacks, the effects of pathogenic and mutualistic fungi have attracted a great deal of attention, suggesting that focal plants can negatively or positively affect the performance of surrounding conspecific and heterospecific individuals by accumulating these soil biota [3, 5, 17, 18]. Logically, this microbe-mediated process is likely a principle for both host-specific and nonspecific fungal species, yet the former ones have been more intensively studied. This is possibly due to the apparent host-specific impacts among plants with a phylogenetically constrained signal [19–21] and the differential effects from generalists across the infected host species (e.g., effective specialization in pathogens) [1, 22]. Largely unknown is the community-level effect from overall root-associated fungal communities (including potential specialists and generalists) and its contribution to the observed plant co-occurrence patterns across highly variable environments. Relating the differentiations of root fungal associations between plant species to their spatial relationships informed from forest inventory data [13] is a promising strategy and provides essential insights into this gap.

Increasing compelling evidence suggests that pathogenic and mutualistic fungi play essential but opposing roles in regulating the plant species diversity and distribution in a large-scale [3, 11, 23, 24], and jointly influence the hosts co-occurrence [17, 18]. The co-occurrence of plants is expected to be dependent on the differences in their sensitivity to pathogens or the benefits they accrue from mutualists. Specifically, if two plant species can be colonized by more shared pathogens, they will experience stronger negative effects when growing closely with each other and are unlikely to co-occur. On the other hand, if they can recruit more shared mutualists, they are likely to experience stronger positive effects, which may promote co-occurrence or alternatively impede co-occurrence because of the enhanced interspecific competition. Thus, our research goal is to explore whether the differentiation of root fungal associations could be considered as a host-dependent proxy of spatial co-occurrence pattern between plants in the species-rich forest ecosystem.

Here, we present a study relating the root-associated fungal communities to the spatial co-occurrence patterns among 39-plant species in a subtropical forest (Fig. S1). First, we estimated the spatial relationships between plant species using spatial statistical approaches [25–27] based on the forest inventory data [28, 29]. Second, we evaluated the community-level host specificity among plant species at different developmental stages [29, 30]. For this purpose, we investigated the fungal communities in 501 root tip samples and 1708 bulk soil samples throughout the 50-ha forest plot with relevant environmental properties. Finally, we related the differentiation of root-associated fungal communities to the observed plant co-occurrence patterns and tested the validity using Random Forest modeling [31]. This study deepens our understanding of the importance of root-associated fungi in plant co-occurrence in subtropical forests.

## Materials and methods

### Brief description

Details related to study site, root tip and background soil sampling, molecular characterization of fungal communities, sequence processing, core root-associated fungal communities, phylogenetic relatedness, functional traits, and spatial co-occurrence patterns of plant species, and spatial variation of soil environmental properties are provided in Supplementary Materials and Methods. To explain the data collection and analyses procedures more clearly, we diagrammed the detailed workflow and key description of the materials and methods in Fig. S1. In addition, the custom R codes underpinning the main analyses and the sample data files are available in figshare (10.6084/m9.figshare.10084625.v5).

Briefly, we collected our soil and root tip samples from a 50-ha typical subtropical forest plot in Heishiding Nature Reserve (111°53′ E, 23°27′ N), located in Guangdong province, China. This plot is one of the large permanent forest plots within a global monitoring network called the Center for Tropical Forest Science-Forest Global Earth Observatory (http://www.forestgeo.si.edu/). The comprehensive plant census of this plot was completed in 2013, and a total of ~218,000 free-standing plant individuals with diameters at breast height (DBH) ≥1 cm were tagged and mapped spatially [29]. In this study, we investigated the co-occurrence among 39-plant species, accounting for ~60% of the total plant individuals across the plot (Table S1 and Fig S2). We divided the plant species into three groups based on their relative abundances: ≥1% (H), 0.1–1% (M), and ≤0.1% (L) [29] (Table S1). We classified the plant individuals into three size classes to represent different plant developmental stages according to their DBH: ≤5 cm for saplings, 5–10 cm for juveniles, and ≥10 cm for adults [29, 30]. We constructed the molecular phylogeny for the plant species using the sequences of four genes (i.e., *rbcL*, *matK*, *ITS1*, and *5.8S*) obtained from GenBank. The pairwise phylogenetic distances of plant species were calculated based on the maximum likelihood phylogenetic tree using the “ape” package (*cophenetic.phylo* function) in R (Table S2). The functional traits of the plant species in this study were downloaded from the TRY website (https://www.try-db.org/) [32] (Table S3). The pairwise functional distance (Gower dissimilarity) was calculated using the “FD” package (*gowdis* function) in R (Table S2). Based on the forest inventory data, we estimated the spatial relationships (i.e., aggregated or segregated) for all the 741 pairs of plant species by applying bivariate pair correlation function *g*_*ij*_(*r*) [25–27] using the “spatstat” package (*pcfcross* functions) in R (Table S4).

In a previous study [29], we randomly collected 529 root tip samples from 45 plant species in the plot (3 to 19 individuals per species with detailed spatial locations recorded from the census data). In this study, to link the spatial patterns between different plants species with their representative root-associated fungal communities, we retained 39-plant species (a total of 501 individuals) with at least 7 replicates for subsequent analyses (Table S1). Meanwhile, we intensively collected a total of 1708 bulk soil samples using a checkerboard design throughout the plot (Fig. S3a). We characterized the root-associated and background soil fungal communities by amplifying and sequencing the second internal transcribed spacer (ITS2) region of fungal rRNA genes following the procedure described previously [29, 33]. Briefly, the entire ITS region was first amplified using the fungal-specific primers ITS1-F (5′-CTTGGTCATTTAGAGGAAGTAA-3′) and ITS4 (5′-TCCTCCGCTTATTGATATGC-3′), and then a second PCR using the primers ITS3 (5′-GCATCGATGAAGAACGCAGC-3′) and ITS4 (5′-TCCTCCGCTTATTGATATGC-3′). For the subsequent analyses, we resampled 3000 and 4100 high-quality sequences for each root tip and background soil sample, respectively. The profiles of root-associated fungi can be influenced by the surrounding soil environment [1, 3, 15], such as background soil properties and fungal communities. Thus, the root fungal associations for a given plant species may vary among individuals, especially across markedly heterogeneous soil environments. To obtain the representative root-associated fungal communities for each plant species, we defined core root-associated fungal OTUs at nine different cutoffs according to their detected frequency (e.g., cutoff = 0.5 indicated that these OTUs could be found in half of the root tip samples of a given plant species). We conducted subsequent analyses using the datasets of the overall OTUs and core OTUs to examine the consistency of the results.

We measured the environmental properties by using the background bulk soil samples (Table S5) and then estimated the spatial variation for the whole 50-ha plot by applying geostatistical interpolation technique of ordinary kriging (*krige* function in “gstat” package in R) (Fig. S3b). Subsequently, the associated soil environmental properties of each plant individuals from which we collected root tip samples were estimated according to their spatial locations (Table S6).

### Host specificity of root-associated fungal communities

We hypothesized that the COMMs of root-associated fungi depend on their hosts and can be differentiated among different plant species. To test this, we compared the root-associated fungal community of each target plant species against every root-associated fungal community of all the other 38 plant species. The community-level host specificity was tested by PERMANOVA test (*Adonis* function, Bray-Curtis distance with permutations = 999, “vegan” package in R) based on the relative abundances of overall OTUs or core OTUs at different cutoffs between every pair of different plant species. For a given plant species, the *P* values from the 38 comparisons were extracted and adjusted by the false discovery rate (*p.adjust* function, method = “fdr”). In this study, we defined the fungal community profile for a given plant species as host-specific when it was significantly (*P* < 0.05) different from those of all the other 38 (i.e., 100%) compared plant species. Meanwhile, we defined the fungal community profile as host-dependent when it was significantly different from those of at least 35 (i.e., >90%) compared plant species. Such host specificity was also tested with the same definition criteria for each plant species at their different developmental stages.

We further validated the host specificity of root-associated fungal communities using environmental properties and fungal communities of background soils in the plot to confirm they were largely dependent on the plant species themselves rather than their background soils. We estimated the environmental properties of each plant individual based on the interpolated environmental properties of background soils as mentioned above (Tables S5, S6). Meanwhile, we estimated the fungal communities (overall OTUs) of surrounding bulk soils by summing up the sequences from the top three nearest background soils related to each plant individual (12,300 reads in total) (Table S7). Notably, the mean distance between the plant individuals and the third nearest bulk soil samples was 14.9 m. To avoid individuals of a given plant species to share the same set of background soils, one of these individuals was randomly selected for subsequent PERMANOVA tests of host-dependent differentiation if they were close to each other with distance <30 m, and a total of 305 root tip samples were then selected (Table S7). Similarly, the host-dependent differentiations of background soil environmental properties and fungal communities were tested with the same definition criteria mentioned above. Furthermore, we conducted Mantel tests (Pearson correlation with permutations of 999 times) to reveal the relationships between the root-associated fungal communities in relation to the background soil environmental properties and fungal communities based on these 305 selected root tip samples, respectively.

### Pairwise differentiation of root fungal associations

We compared the root-associated fungal communities of each pair of plants. We aimed to explore whether such pairwise differentiation of root fungal associations could be used to (i) approximate the community-level effects on the outcome of potential plant-soil feedbacks when plants are growing together and interacting, and (ii) subsequently reflect the spatial co-occurrence of host plants.

For a plant pair, we split the root-associated fungal species into shared (i.e., detected in both plants) and unique (i.e., detected in either plant) core OTUs to differentiate the influences from potential, pairwise “generalists” and “specialists”, respectively. We then calculated an index (termed “modified relative abundance”) to evaluate the relative abundance of each shared (or unique) core fungal OTU (an example of the calculation procedure are shown in Fig. S4). Specifically, for the MRAs of unique core OTUs, we divided their relative abundances by the sum of relative abundances from all unique core OTUs. For the MRAs of shared core OTUs, we divided their products of relative abundances by the sum of each product between the two relative abundances of shared core OTUs. Thus, for a pair of plants, the sum of MRAs of all shared (or unique) core fungal OTUs was adjusted to 1. A fungal OTU had a higher value of MRA implied higher recruitment of this fungal species when plants grew together.

We applied this calculation procedure for all pairs of different plant species and the pairs of plant species at their different developmental stages. In addition, we calculated these profiles of MRAs based on the core OTUs at cutoffs of 0.5 and 0.9. We obtained the MRA profiles for shared (or unique) OTUs and used them as the input predictor variables in the subsequent Random Forest modeling.

### Prediction of spatial relationships between plant species

By applying Random Forest modeling (classification procedure), a machine learning approach [31], we predicted the spatial co-occurrence patterns (i.e., aggregated or segregated) of all pairs of 39-plant species based on different predictive variables, including the species-level dissimilarities of relative abundance (RA), phylogeny (PL), functional trait (FT), richness (RICH), and community composition (COMM) of overall root-associated fungal OTUs between plants (Table S2), and the modified relative abundance (MRA) profiles of shared (or unique) core root-associated fungal OTUs at cutoffs of 0.5 and 0.9. Modeling analyses were also conducted using the MRA profiles for plant species at their different developmental stages. Notably, we did not test the importance of environmental properties in this study because they were interpolated based on the spatial autocorrelation, and were not suitable for testing our spatial co-occurrence hypothesis.

We developed rules to assign plant pairs into two classes (i.e., aggregated and segregated) and to assess the performing differences among different predictor variables. We used one single predictor variable (i.e., the species-level dissimilarity) in five different models to examine how the differences of RA (or PL, FT, RICH, COMM) between plant species can predict the pairwise spatial relationships. Meanwhile, we considered each of the shared (or unique) core root-associated fungal OTUs as a separate predictor in the models based on the MRA profiles (Fig. S4). For each model, 80% of the total dataset was randomly selected for training, and the remaining 20% of the dataset was used for validation. For the training dataset, we used the “ROSE” package (*ovun.sample* function in R) to deal with binary classification problems in the presence of imbalanced data. Simultaneously, we generated a shuffled dataset for null model testing by randomizing the labels associated with the real training dataset. Then we performed the Random Forest modeling using both observed and shuffled datasets by the “randomForest” package (*randomForest* function, trained with 1000 trees). After model construction, the receiver operating characteristic (ROC) curves were computed based on the associated validation dataset, and the area under the ROC curve (AUC) was calculated using the “ROCR” package (*prediction* and *performance* functions). To assess the modeling performance, we repeated the whole process of modeling 100 times and obtained the AUC values from both observed and shuffled data for all different dissimilarities of plant features. For a random guess, the AUC value is near 0.5. The performance differences between models using observed and shuffled data were compared by Wilcoxon tests and the significances (*P* values) were adjusted by the false discovery rate (Benjamini algorithm). One-way ANOVA (ANalysis Of VAriance) with post hoc Tukey HSD (Honestly Significant Difference) test was conducted using the “agricolae” package (*aov* and *HSD.test* functions) to compare the distributions of AUC values for different predictor variables.

Finally, we summarized the modified RAs of core OTUs (cutoff = 0.5) for different functional guilds and examine their differences between plant pairs with distinct spatial co-occurrence patterns. The putative functionality (e.g., fungal functional guilds) of core OTUs was determined by searching against the fungal database program FUNGuild [34] based on their taxonomic affinity, following the approach that has been widely applied in recent studies [10, 11, 28, 35]. In this study, we specifically focused on the core OTUs that could be annotated into functional guilds as “plant pathogen”, “ectomycorrhizal (EcM) fungi”, or “saprotrophs”. The modified RAs of these putative functional guilds were calculated as the sum of modified RAs from the shared (or unique) core OTUs that could be assigned to them. Core OTUs that had multiple function assignments in FUNGuild were excluded from the analysis. We explored the functional differentiation by comparing the modified RAs of fungal functional guilds between aggregated and segregated plant pairs at different developmental stages using Wilcoxon tests (*wilcox_test* and *wilcox_effsize* functions in “rstatix” package).

## Results

### Plant features and root-associated fungi

**Fig. 1:**
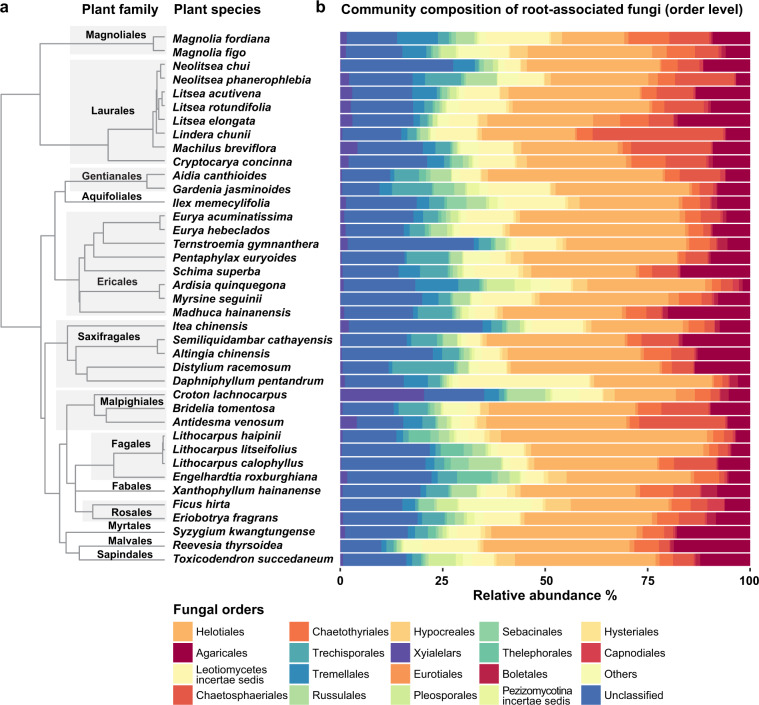
Molecular phylogeny and community composition of root-associated fungi for the 39-plant species in this study. **a** The maximum likelihood phylogenetic tree that was constructed for the selected 39-plant species using the sequences of four genes (i.e., *rbcL*, *matK*, *ITS1*, and *5.8S*) that obtained from GenBank. **b** Mean relative abundances % of different root fungal orders that were associated with each plant species. The most dominant fungal orders were affiliated with Helotiales (30.7%), Agaricales (9.5%), Leotiomycetes incertae sedis (8.1%), Chaetosphaeriales (7.4%), Chaetothyriales (4.5%), Trechisporales (4.2%), Tremellales (2.8%), Russulales (2.6%), Hypocreales (1.9%), Xylariales (1.6%), and Eurotiales (1.3%) collectively accounting for ~75% of the total sequencing reads.

The 39 investigated plant species accounted for ~60% of the total plant individuals across the 50-ha subtropical forest plot (Table S1), which represents a wide range of RA, phylogenetic relatedness, and FTs (Fig. 1; Tables S2, S3). Our spatial statistic results showed distinct co-occurrence patterns between different pairs of plant species (Table S4) in this markedly heterogeneous soil environment (Figs. S3, S5; Tables S5, S6).

We identified a total of 11,720 fungal OTUs from the root tip samples among the 39-plant species, revealing high variations in root-associated fungal diversity and COMM (Fig. 1; Tables S1, S2, S8). In addition, we found a marginal correlation between the overall fungal community and the host PL (Mantel test: *r* = 0.062, *P* = 0.056), implying that they might be decoupled. This result confirmed, to our expectation, that the root-associated fungal communities could be host-specific and may be independent of phylogenetic relatedness between plants.

### Host-dependent fungal associations

To test the host specificity, we defined core OTUs for each plant species to obtain the representative root-associated fungal communities. The number of core root-associated fungal OTUs among the nine different cutoffs (i.e., detected frequency) ranged from 683 to 269, with an average of 212 ± 14 to 135 ± 8 (mean ± s.d.) per plant species (Fig. S6). Although the core fungal species richness dramatically decreased when considering more frequently detected OTUs, they represented, on average, 95% (at cutoff = 0.5) to 86% (at cutoff = 0.9) of the total sequencing reads per plant species (Fig. S6), indicating high representativeness of plant root-associated fungal communities.

**Fig. 2:**
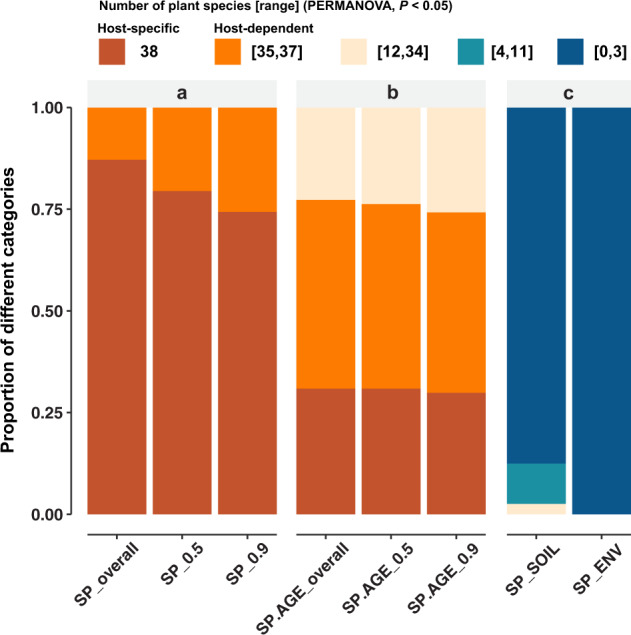
Host specificity of root-associated fungal community. PERMANOVA tests were applied to examine the differences of root-associated fungal communities between every pair of different plant species based on the relative abundances of overall or core OTUs (cutoffs of 0.5 and 0.9 are shown as examples). **a** For each target plant species (SP, *n* = 39), comparisons were conducted separately against all the other 38 plant species. Different categories show the ranges of the number of plant species with significant (*P* < 0.05) difference. The fungal community profile for a given plant species was defined as host-specific when it was significantly different from those of all the other 38 (i.e., 100%) compared plant species. Meanwhile, it was defined as host-dependent when it was significantly different from those of at least 35 (i.e., >90%) compared plant species (also see Table S9). **b** Host specificity was tested with the same definition criteria for each plant species at their different life stages (SP.AGE, *n* = 97, also see Table S10). **c** Validation of the host specificity of root-associated fungal communities (SP, *n* = 39) was conducted based on the fungal communities (SOIL) and environmental properties (ENV) of background soils in the plot (also see Table S11).

Based on the RAs of overall or core OTUs, we tested the community-level host specificity by comparing the root-associated fungal community of each target plant species against those of all the other 38 plant species (PERMANOVA test). We defined the fungal community as host-specific or host-dependent when it was significantly (*P* < 0.05) different from those of 38 (i.e., 100%) or at least 35 (i.e., >90%) compared plant species, respectively. Our results indicated that the fungal communities of all 39-plant species were host-dependent, with 29 of them (~74%) were host-specific (Fig. 2a; Table S9). Since the root-associated fungal COMM could change during the plant growth, we examined the host specificity by considering plant developmental stages as well, and still found such host-dependent differentiations of fungal communities in ~75% of the plants (*n* = 97) at a certain developmental stage (Fig. 2b; Table S10). Moreover, we observed that this host-dependency tended to be stronger in larger trees (adults and juveniles), as the proportion of nonspecific profiles in adults (*n* = 27), juveniles (*n* = 36), and saplings (*n* = 34) were 26%, 19%, and 32%, respectively.

To test whether the nonrandom patterns of root-associated fungal communities were caused by the soil environmental or local fungal community differences across space, we further validated the host specificity using the background soil environmental properties and fungal communities (Tables S6, S7). Our results demonstrated that plant species were not located at specific habitats with significant differences in environmental properties and fungal communities from the background soils (Fig. 2c; Table S11). Moreover, the Mantel tests showed that the root-associated fungal communities were not significantly (*P* > 0.05) related to the background soil environmental properties or fungal communities, even though the environmental properties and bulk soil fungal communities were significantly correlated (*r* = 0.13, *P* = 0.001) (Table S12). These results suggested that the host-dependent differentiation of root-associated fungal communities largely depended on the characteristics of the plant hosts, and not the soil environment. The relatively stronger host dependency in larger trees further offered a clue about the potential process of how plants gradually form this specific association as they grow with diverse surrounding fungal communities.

### Differentiation of root-associated fungi and plant co-occurrence

**Fig. 3:**
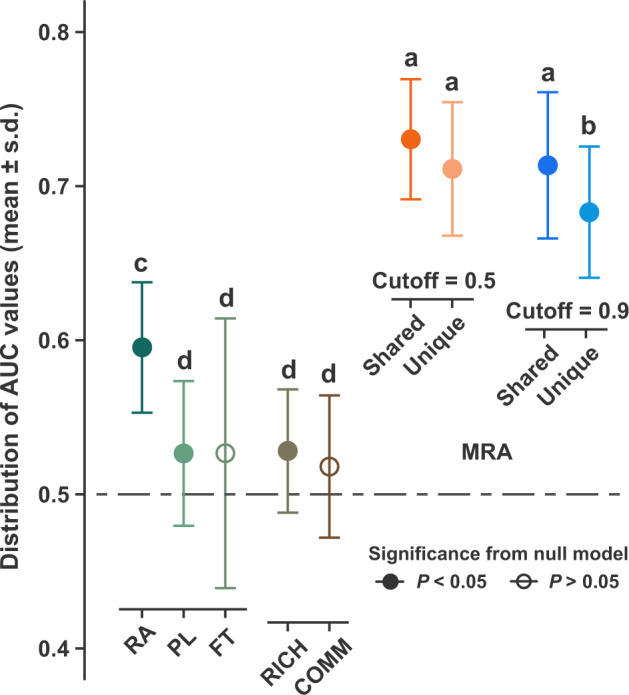
Prediction performance of spatial co-occurrence pattern using different dissimilarities of plant features. Random Forest modeling was conducted based on different predictor variables separately, including the dissimilarities of relative abundance (RA), phylogeny (PL), functional trait (FT), richness (RICH), and community composition (COMM) of overall fungal OTUs, and the modified relative abundance (MRA) profiles of shared (or unique) core root-associated fungal OTUs at cutoffs of 0.5 and 0.9. The performance differences between models using observed and shuffled data were compared by Wilcoxon tests. One-way ANOVA with post hoc Tukey HSD test was conducted to compare the distributions of AUC values for different predictor variables. Prediction performance of different predictor variables with the same letter were not significantly different at alpha value of 0.05.

By applying Random Forest modeling, we found that the modified RAs for core root-associated fungal OTUs showed significantly higher (ANOVA with Turkey HSD test) prediction performance among the predictor variables (Fig. 3), indicating higher classification ability to assign a plant pair into a correct, observed co-occurrence state (i.e., spatially aggregated or segregated). Moreover, our results revealed that such significantly higher prediction accuracy by using the modified RAs was consistent for shared and unique core fungal OTUs and across different developmental stages of plants (Fig. 3; Table S13). However, we observed that extremely constrained definition (e.g., cutoff = 0.9) of core fungal species in the unique profile substantially decreased the prediction performance (Table S13), implying the non-ignorable effects from some potential “specialists” with relatively lower infection rates. In general, our analysis indicated that the host-dependent differentiation of root-associated fungal communities could increase our ability to explain the spatial co-occurrence patterns.

**Fig. 4:**
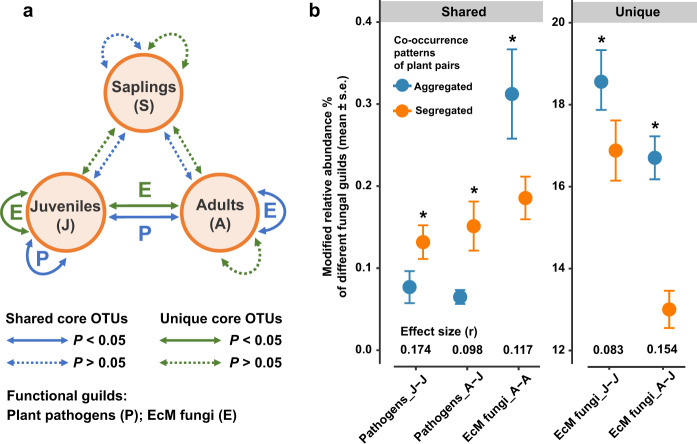
Functional differentiation between spatially aggregated and segregated plant pairs at different developmental stages. **a** Functional guilds with significant (*P < 0.05) difference between aggregated and segregated plant pairs at different developmental stages. **b** The modified relative abundance (%, mean ± s.e.) and effect size (r) for functional guilds with significant (*P < 0.05) difference between aggregated and segregated plant pairs at different developmental stages (also see Table S14). Wilcoxon tests were conducted based on the modified relative abundances (%) of three putative functional guilds, including “plant pathogen”, “ectomycorrhizal (EcM) fungi”, and “saprotrophs“. The modified relative abundances of these putative fungal guilds were calculated as the sum of relative abundances from the shared (or unique) core OTUs that could be assigned to them. Core OTUs that had multiple function assignments in FUNGuild were excluded from the analysis.

We examined the functional differentiation of root-associated fungi (based on the modified RAs of different functional guilds) between the spatially aggregated and segregated plant pairs and related it to the co-occurrence patterns across different plant developmental stages. Among the 683 core fungal species, 244 of them could be assigned to different functional guilds, accounting for 26.6% of the total sequences. By exploring the putative functional guilds including plant pathogen, ectomycorrhizal (EcM) fungi, and saprotrophs, we found significant differences in the modified RAs of the pathogens and EcM fungi between spatially aggregated and segregated plant pairs only at their later developmental stages (i.e., juveniles and adults) (Fig. 4a; Table S14). Nevertheless, the modified RAs of saprotrophs remained similar and showed no significant difference (Table S14). Statistically, the shared pathogens had significantly higher modified RAs for the pairs of plants species that were spatially segregated, while the modified RAs of unique EcM fungi was significantly higher for spatially aggregated plant pairs (Fig. 4b). These negative and positive effects via accumulating pathogens and mutualists implied two potential mechanisms underlying the plant coexistence.

## Discussion

**Fig. 5:**
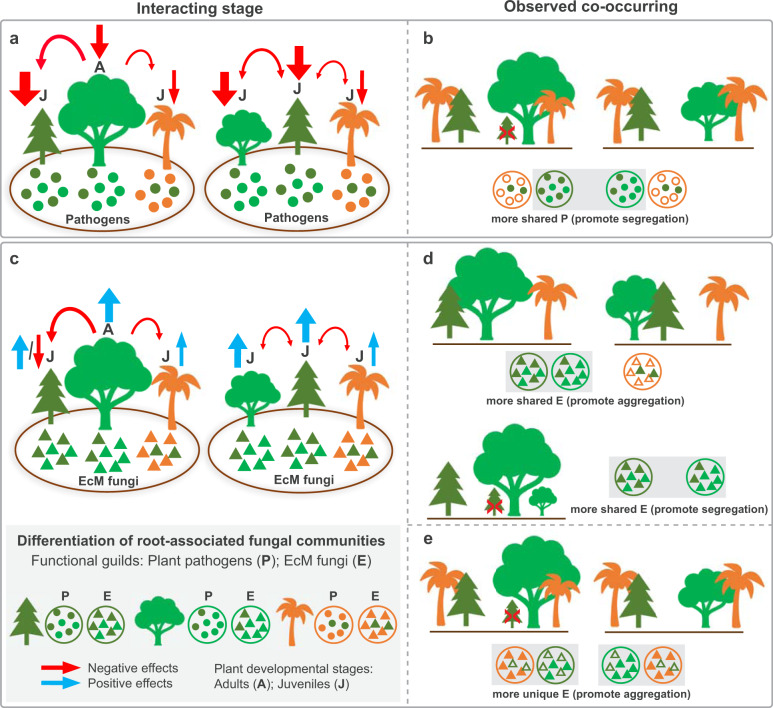
A conceptual diagram illustrating how potential negative and positive effects from root-associated fungi reflect the co-occurrence patterns of host plants in the field. The effects from root-associated fungi may cause differential interactions between plants at their different developmental stages (**a**, **c**) resulting in different observed co-occurrence patterns (**b**, **d**, **e**). In this diagram, the profiles of root-associated fungal communities are differentiated by plants and their magnitude of similarity by colors; as the dark and light green plants share more similar fungal species while the orange plant is infected by more unique fungi. Additionally, the strength of the effect from the fungal species is represented by the thickness of the arrows, with red and blue arrows indicate negative and positive effects, respectively. The size of the plants represents their different developmental stages.

Our findings provide evidence that the established trees likely harbor a distinctive consortium of fungi in roots under natural, highly variable environments. This community-wide host dependency is increasingly acknowledged in recent surveys of root-associated fungal assemblage, such as those among tree species in neotropical [16] and subtropical [36] forests, and *Burmannia* plants with different trophic modes [37]. In addition, the patterns of host dependency are observed in different fungal guilds (e.g., pathogenic and mycorrhizal fungi) [16, 36]. This host-dependent association is probably due to the plant’s specialized traits and its evolutionary history with the fungal partners [3], which helps to explain the observed strong relationship linking the plant species identity and diversity to the belowground fungal communities [35, 38, 39]. In this study, we further expect host-dependent recruitment of belowground fungal communities, which may lead to predictable plant-plant interactions and reflect the observed co-occurrence patterns of diverse plant species. Our results reveal consistent trends that negative and positive effects via accumulating pathogens and mutualists can promote spatial segregation and aggregation, respectively. While the differences in the strength of these two relationships (see effect size in Fig. 4b) provide some important clues that may help to disentangle the potential primary mechanisms when considering the interactions between plants at their different developmental stages (Fig. 5).

For the negative effects in the coexistence between heterospecific plants (Fig. 5a), the classic heterospecific negative density dependence suggests that the pressure from generalist pathogens or a stronger interspecific competition can result in a suppression of establishment and growth of heterospecific individuals [40]. Although generalist fungal pathogens usually have differential effects on plant fitness across the infected host species [1, 22], the accumulation of these pathogenic generalists will probably reduce the performance of their hosts and can cause the replacement of inferior competitors [5]. As such, stronger negative effects will impose on those interacting trees that could be infected with more abundant shared pathogens and lead to a greater competitive exclusion (Fig. 5b, left panel) and/or habitat partitioning/species sorting (Fig. 5b, right panel). This mechanism via the recruitment of generalist pathogens supports our observations (Fig. 4b, Shared), providing a possible explanation of the apparent spatial segregation between the light and dark green trees in Fig. 5b at different developmental stages.

Alternatively, it is well documented that the EcM fungi benefit host plants by providing substantial protection against pathogens and enhancing their nutrient access and stress tolerance [3], and commonly lead to monodominant plant communities through positive feedbacks [41]. How these positive effects influencing the heterospecific co-occurrence patterns may depend on the strength of the intraspecific and heterospecific interactions. For example, the light and dark green trees can harbor more shared, ‘generalist’ EcM fungi (Fig. 5c), and the strength of intraspecific promotion is not stronger than the heterospecific one. In this case, we may expect stronger net positive effects promote their aggregation (Fig. 5d, upper panel) because the symbiotic associations can enhance their growth performance and offer them a safe environment from pathogens [11, 42], especially at the early developmental stage. In contrast, weaker positive or even net negative effects may be expected when the intraspecific promotion is much stronger, which can lead to monodominance and cause their segregation due to the increase of interspecific competition (Fig. 5d, lower panel). Instead of supporting these expectations, our results (Fig. 4b, Unique) highlight that hosting more abundant unique, ‘specialist’ EcM fungi may facilitate the spatial aggregation (e.g., orange tree in Fig. 5c, e), implying an important strategy for driving the clustering, particularly between early established trees and the heterospecific adults (Fig. 4b, Effect size). Furthermore, our findings support the idea that increasing biotic niche differentiation via partner specificity can help to reduce interspecific competition relative to intraspecific competition and hence enhance coexistence [3, 43].

Our results also showed that the modified RAs of shared EcM fungi were significantly higher for spatially aggregated adults rather than segregated ones (Fig. 4b, Shared, EcM fungi_A-A). This spatial aggregation between interspecific adults may result from a net positive effect via accumulating shared EcM fungi as mentioned above (i.e., Fig. 5d, upper panel). However, the microbe-driven processes may not be sufficient to regulate the survival of long-term co-occurring adults. In this case, we considered that this pattern might be due to the lesser specific belowground fungal linkages between plant species [44], which may help redistribute carbon and nutrients among plants, regulate competition [45], and thus maintain coexistence.

Incorporating belowground microbial communities into plant population dynamics has been advocated for two decades [46, 47]. Current trends in research continue to advance our understanding of the theoretical foundation for how plant-microbe interactions influence plant diversity [6, 48]. Moreover, new FTs such as nutrient-acquisition strategies [9, 10], mycorrhizal type [8, 11], and plant transcriptomics [49] have been identified to explain how different plants respond to the changes in soil biota or environmental properties and co-occur with one another. In this study, our analyses support that the root-associated communities are host-dependent. Furthermore, our findings suggest that distinguishing the differentiation of these fungal associations is helpful in estimating the outcome of potential plant-soil feedbacks and the field-based plant co-occurrence patterns. Nevertheless, the results from our observational study need to be evaluated by comprehensive experiments with control settings. Moreover, the host dependency of root-associated fungal communities among more diverse species across more distinctive habitats needs to be validated in the future. We also acknowledge that the basis of the fungal functional interpretation relies on the current database (e.g., FUNGuild), and the functionality of many unassigned and multi-assigned OTUs is unclear or changes at different conditions. Thus, empirical data are required to examine the generality of the relationships observed here.

Notably, although some of the target plant species are likely associated with arbuscular mycorrhizal (AM) fungi [14], the communities of AM fungi are not well characterized in this study, and their effects on the spatial relationships between plant species remain unclear. Recent studies have reported that the primers we used are unsuitable for amplifying AM fungi (from the phylum Glomeromycota) [50]. We admit that our results cannot provide a complete understanding of the roles of AM and EcM fungi in mediating plant co-occurrence due to this possible PCR bias. It is commonly recognized that AM plants experience more negative soil feedback from their adults compared with EcM plants [3, 8]. Thus, AM plants would be likely self-limiting due to the enhance of intraspecific competition [8, 51]. As a result, this negative plant-soil feedback in the AM system likely suppresses superior competitors and alleviates interspecific competition, promoting plant coexistence [3]. Nevertheless, how the pairwise differentiation of AM fungal communities explains the spatial co-occurrence patterns needs to be examined, and the new general and AM fungi-specific primer sets help to improve the completeness of fungal diversity [52–54].

In conclusion, we demonstrate that the root-associated fungal community was host-dependent among 39-plant species in a 50-ha subtropical forest plot. Discriminating the pairwise differentiation of these fungal associations can significantly increase our ability to explain spatial co-occurrence patterns. Recent advances in next‐generation sequencing unprecedentedly strengthen our ability to recover the biodiversity in Earth mycobiome [2], and a huge database of root-associated fungal communities is rapidly accumulating. Given that the root fungal “fingerprinting” is a putative host-dependent plant feature, incorporating it into the plant diversity pattern studies can extend our understanding of plant–plant interactions and will be useful in assessing the invasion success of exotic plants in local species-rich communities.

## Supplementary information

Supplementary Materials and Methods

Supplementary Figures

Table S1

Table S2

Table S3

Table S4

Table S5

Table S6

Table S7

Table S8

Table S9

Table S10

Table S11

Table S12

Table S13

Table S14

## Data Availability

The custom R codes underpinning the main analyses and the sample data files, as well as the representative sequences of fungal OTUs and their abundances for root tip and background soil samples are available in figshare (10.6084/m9.figshare.10084625.v4).

## References

[1] Bever JD, Mangan SA, Alexander HM (2015). Maintenance of plant species diversity by pathogens. Annu Rev Ecol Evol Syst.

[2] Peay KG (2016). The mutualistic niche: mycorrhizal symbiosis and community dynamics. Annu Rev Ecol Evol Syst.

[3] Tedersoo L, Bahram M, Zobel M (2020). How mycorrhizal associations drive plant population and community biology. Science..

[4] Bever JD, Dickie IA, Facelli E, Facelli JM, Klironomos J, Moora M (2010). Rooting theories of plant community ecology in microbial interactions. Trends Ecol Evol.

[5] Bever JD, Platt TG, Morton ER (2012). Microbial population and community dynamics on plant roots and their feedbacks on plant communities. Annu Rev Microbiol.

[6] Ke PJ, Miki T (2015). Incorporating the soil environment and microbial community into plant competition theory. Front Microbiol.

[7] Mangan SA, Schnitzer SA, Herre EA, Mack KM, Valencia MC, Sanchez EI (2010). Negative plant-soil feedback predicts tree-species relative abundance in a tropical forest. Nature.

[8] Bennett JA, Maherali H, Reinhart KO, Lekberg Y, Hart MM, Klironomos J (2017). Plant-soil feedbacks and mycorrhizal type influence temperate forest population dynamics. Science..

[9] Teste FP, Kardol P, Turner BL, Wardle DA, Zemunik G, Renton M (2017). Plant-soil feedback and the maintenance of diversity in Mediterranean-climate shrublands. Science..

[10] Semchenko M, Leff JW, Lozano YM, Saar S, Davison J, Wilkinson A (2018). Fungal diversity regulates plant-soil feedbacks in temperate grassland. Sci Adv.

[11] Chen L, Swenson NG, Ji N, Mi X, Ren H, Guo L (2019). Differential soil fungus accumulation and density dependence of trees in a subtropical forest. Science.

[12] LaManna JA, Walton ML, Turner BL, Myers JA (2016). Negative density dependence is stronger in resource-rich environments and diversifies communities when stronger for common but not rare species. Ecol Lett.

[13] Eppinga MB, Baudena M, Johnson DJ, Jiang J, Mack KM, Strand AE (2018). Frequency-dependent feedback constrains plant community coexistence. Nat Ecol Evol.

[14] Brundrett MC (2002). Coevolution of roots and mycorrhizas of land plants. New Phytol.

[15] van der Linde S, Suz LM, Orme CDL, Cox F, Andreae H, Asi E (2018). Environment and host as large-scale controls of ectomycorrhizal fungi. Nature..

[16] Schroeder JW, Martin JT, Angulo DF, Razo IAD, Barbosa JM, Perea R (2019). Host plant phylogeny and abundance predict root‐associated fungal community composition and diversity of mutualists and pathogens. J Ecol.

[17] Jiang J, Karen CA, Mara B, Maarten BE, James AE, James DB (2020). Pathogens and mutualists as joint drivers of host species coexistence and turnover: implications for plant competition and succession. Am Nat.

[18] Schroeder JW, Dobson A, Mangan SA, Petticord DF, Herre EA (2020). Mutualist and pathogen traits interact to affect plant community structure in a spatially explicit model. Nat Commun.

[19] Gilbert GS, Webb CO (2007). Phylogenetic signal in plant pathogen‐host range. Proc Natl Acad Sci USA.

[20] Liu X, Liang M, Etienne RS, Wang Y, Staehelin C, Yu S (2012). Experimental evidence for a phylogenetic Janzen‐Connell effect in a subtropical forest. Ecol Lett.

[21] Liang M, Liu X, Etienne RS, Huang F, Wang Y, Yu S (2015). Arbuscular mycorrhizal fungi counteract the Janzen‐Connell effect of soil pathogens. Ecology..

[22] Benítez MS, Hersh MH, Vilgalys R, Clark JS (2013). Pathogen regulation of plant diversity via effective specialization. Trends Ecol Evol.

[23] Klironomos J, Zobel M, Tibbett M (2011). Forces that structure plant communities: quantifying the importance of the mycorrhizal symbiosis. New Phytol.

[24] van der Heijden MGA, Bardgett RD, van Straalen NM (2008). The unseen majority: soil microbes as drivers of plant diversity and productivity in terrestrial ecosystems. Eco Lett.

[25] Wiegand T, Moloney KA (2004). Rings, circles, and null‐models for point pattern analysis in ecology. Oikos..

[26] Perry GL, Miller BP, Enright NJ (2006). A comparison of methods for the statistical analysis of spatial point patterns in plant ecology. Plant Ecol.

[27] Law R, Illian J, Burslem DF, Gratzer G, Gunatilleke CV, Gunatilleke IA (2009). Ecological information from spatial patterns of plants: insights from point process theory. J Ecol.

[28] Liang M, Liu X, Parker IM, Johnson D, Zheng Y, Luo S (2019). Soil microbes drive phylogenetic diversity-productivity relationships in a subtropical forest. Sci Adv.

[29] Chen Y, Jia P, Cadotte MW, Wang P, Liu X, Qi Y (2019). Rare and phylogenetically distinct plant species exhibit less diverse root-associated pathogen communities. J Ecol.

[30] Peters HA (2003). Neighbour‐regulated mortality: the influence of positive and negative density dependence on tree populations in species‐rich tropical forests. Ecol Lett.

[31] Cutler DR, Edwards TC, Beard KH, Cutler A, Hess KT, Gibson J (2007). Random forests for classification in ecology. Ecology..

[32] Kattge J, Diaz S, Lavorel S, Prentice IC, Leadley P, Bönisch G (2011). TRY - a global database of plant traits. Glob Chang Biol..

[33] Davey ML, Heegaard E, Halvorsen R, Ohlson M, Kauserud H (2012). Seasonal trends in the biomass and structure of bryophyte-associated fungal communities explored by 454 pyrosequencing. New Phytol.

[34] Nguyen NH, Song Z, Bates ST, Branco S, Tedersoo L, Menke J (2016). FUNGuild: an open annotation tool for parsing fungal community datasets by ecological guild. Fungal Ecol.

[35] Leff JW, Bardgett RD, Wilkinson A, Jackson BG, Pritchard WJ, Jonathan R (2018). Predicting the structure of soil communities from plant community taxonomy, phylogeny, and traits. ISME J.

[36] Wang Z, Jiang Y, Deane DC, He F, Shu W, Liu Y (2019). Effects of host phylogeny, habitat and spatial proximity on host specificity and diversity of pathogenic and mycorrhizal fungi in a subtropical forest. New Phytol.

[37] Zhao Z, Li X, Liu MF, Merckx VS, Saunders RM, Zhang D (2021). Specificity of assemblage, not fungal partner species, explains mycorrhizal partnerships of mycoheterotrophic *Burmannia* plants. ISME J.

[38] Peay KG, Baraloto C, Fine PV (2013). Strong coupling of plant and fungal community structure across western Amazonian rainforests. ISME J.

[39] Barberán A, McGuire KL, Wolf JA, Jones FA, Wright SJ, Turner BL (2015). Relating belowground microbial composition to the taxonomic, phylogenetic, and functional trait distributions of trees in a tropical forest. Ecol Lett.

[40] LaManna JA, Belote RT, Burkle LA, Catano CP, Myers JA (2017). Negative density dependence mediates biodiversity-productivity relationships across scales. Nat Ecol Evol.

[41] Peh KS, Lewis SL, Lloyd J (2011). Mechanisms of monodominance in diverse tropical tree‐dominated systems. J Ecol.

[42] Johnson DJ, Clay K, Phillips RP (2018). Mycorrhizal associations and the spatial structure of an old-growth forest community. Oecologia..

[43] Waud M, Busschaert P, Lievens B, Jacquemyn H (2016). Specificity and localised distribution of mycorrhizal fungi in the soil may contribute to co-existence of orchid species. Fungal Ecol.

[44] Põlme S, Bahram M, Jacquemyn H, Kennedy P, Kohout P, Moora M (2018). Host preference and network properties in biotrophic plant–fungal associations. New Phytol.

[45] Simard SW, Beiler KJ, Bingham MA, Deslippe JR, Philip LJ, Teste FP (2012). Mycorrhizal networks: mechanisms, ecology and modelling. Fungal Biol Rev.

[46] Bever JD, Westover KM, Antonovics J (1997). Incorporating the soil community into plant population dynamics: the utility of the feedback approach. J Ecol.

[47] Bardgett RD, Wardle DA. Aboveground-belowground linkages: biotic interactions, ecosystem processes, and global change. New York: Oxford University Press; 2010.

[48] Kandlikar GS, Johnson CA, Yan X, Kraft NJ, Levine JM (2019). Winning and losing with microbes: how microbially mediated fitness differences influence plant diversity. Ecol Lett.

[49] Swenson NG, Iida Y, Howe R, Wolf A, Umaña MN, Petprakob K (2017). Tree co-occurrence and transcriptomic response to drought. Nat Commun.

[50] Řezáčová V, Gryndler M, Bukovská P, Šmilauer P, Jansa J (2016). Molecular community analysis of arbuscular mycorrhizal fungi—contributions of PCR primer and host plant selectivity to the detected community profiles. Pedobiologia..

[51] Hart MM, Reader RJ, Klironomos JN (2003). Plant coexistence mediated by arbuscular mycorrhizal fungi. Trends Ecol Evol.

[52] Taylor DL, Walters WA, Lennon NJ, Bochicchio J, Krohn A, Caporaso JG (2016). Accurate estimation of fungal diversity and abundance through improved lineage-specific primers optimized for Illumina amplicon sequencing. Appl Environ Microbiol.

[53] Lekberg Y, Vasar M, Bullington LS, Sepp SK, Antunes PM, Bunn R (2018). More bang for the buck? Can arbuscular mycorrhizal fungal communities be characterized adequately alongside other fungi using general fungal primers?. New Phytol.

[54] Egan CP, Rummel A, Kokkoris V, Klironomos J, Lekberg Y, Hart MM (2018). Using mock communities of arbuscular mycorrhizal fungi to evaluate fidelity associated with Illumina sequencing. Fungal Ecol.

