# Supplementary material for: Root-associated fungal community reflects host spatial co-occurrence patterns in a subtropical forest
Source: ISME Commun. 2021 Nov 6;1:65. doi: 10.1038/s43705-021-00072-6 (PMC9723750; doi:10.1038/s43705-021-00072-6)
Supplement: Supplementary file 1 — Supplementary Materials and Methods [file 43705_2021_72_MOESM1_ESM.docx]

**Root-associated fungal community reflects host spatial co-occurrence patterns in a subtropical forest**

Jialiang Kuang*, Shun Han, Yongjian Chen, Colin T. Bates, Pandeng Wang, Wensheng Shu^*^

**Supplementary Materials and Methods**

**Study site**

We collected our soil and root tip samples from a 50-ha forest plot in Heishiding Nature Reserve (111°53′ E, 23°27′ N), located in Guangdong province, China. This plot is one of the large permanent forest plots within a global monitoring network called the Center for Tropical Forest Science-Forest Global Earth Observatory (CTFS-ForestGEO) (<http://www.forestgeo.si.edu/)>. This is a typical subtropical evergreen broad-leaved forest with moist monsoon climate. The mean maximum temperature is 19.6°C, and monthly temperatures range from 10.6°C in January to 28.4°C in July. The mean annual precipitation is about 1,743.8 mm. The plot elevation ranges from 435.4 m to 698.4 m and the slope varies from 6.89° to 74.6°. The census of this plot was completed in 2013, and a total of ~218,000 free-standing plant individuals (including 71 families, 160 genera, and 245 species) with diameters at breast height (DBH) ≥ 1cm were tagged and mapped spatially.

**Root tip sampling**

In a previous study [29], we randomly collected 529 root tip samples from 45 plant species in the plot (3 to 19 individuals per species with detailed spatial locations recorded from the census data), representing wide ranges of both abundance and phylogenetic relatedness of the overall plant community (Fig. 1; Table S1). The collection of root tip samples was assisted by the experts of plant taxonomy and morphology from the research group of Prof. Fangliang He (PI of the Heishiding 50-ha plot, https://forestgeo.si.edu/sites/asia/heishiding). To confirm that the sampled root tips were from each of our target individuals, we first identified the primary root and then partially excavated three root tip samples from each individual. We carefully removed the adhering soil and plant debris on root tips. The root tip samples were stored in sterilized plastic bag and immediately transported to the lab on ice for DNA extraction. In this study, to link the spatial patterns between different plants species with their representative root-associated fungal communities, we retained 39 plant species (a total of 501 individuals) with at least 7 replicates for subsequent analyses (Table S1). These selected 39 plant species accounted for ~60% of the total plant individuals across the plot (Fig. S2) and were divided into three groups based on their relative abundances: ≥1% (H), 0.1%~1% (M), and ≤0.1% (L) [29] (Table S1). In addition, we classified the selected plant individuals into three size classes to represent plant developmental stages according to their DBH: ≤5 cm for saplings, 5-10 cm for juveniles, and ≥10 cm for adults [29, 30].

**Background soil sampling**

In August 2013, a checkerboard of non-overlapping regular grid cells (30 × 30 m) was constructed in the plot. We randomly selected 427 grid points for subsequent study, accounting for approximately 74% of all 578 grid points. We collected background soil samples from the selected grid points and additional sampling points that were designated 2m, 4m, and 12m away from each selected grid point along a randomly selected compass direction (Fig. S3a). For each sampling point, its spatial location was recorded, and three 2cm diameter adjacent soil cores (0-10 cm depth) were collected, mixed together in the field and immediately kept on ice for transportation to the laboratory. A total of 1,708 bulk soil samples distributed throughout the plot (Fig. S3a) were collected and sieved to remove roots and stones. A subsample of 0.5g soil was stored at -20^o^C before DNA extraction.

**Molecular characterization of fungal communities**

The DNA of root tip samples was extracted by the E.N.Z.A.^®^ HP Plant DNA Extraction Kit (Omega BioTek, Doraville, USA) following the product instructions. Background soil DNA was extracted using the MoBio PowerSoil DNA extraction kit (Carlsbad, CA) following the manufacturer’s instructions. Before the DNA extraction of root tip samples, all three root tips for each plant individual were washed with running distilled water and ground using liquid nitrogen to mix them as a composite sample [29, 55]. To characterize the root-associated and background soil fungal communities, the second internal transcribed spacer (ITS2) region of fungal rRNA genes was amplified by nested PCR following the procedure described previously [29, 33]. Briefly, the entire ITS region was first amplified using the fungal-specific primers ITS1-F (5’-CTTGGTCATTTAGAGGAAGTAA-3’) and ITS4 (5’- TCCTCCGCTTATTGATATGC-3’), and then a second PCR using the primers ITS3 (5’- GCATCGATGAAGAACGCAGC-3’) and ITS4 (5’-TCCTCCGCTTATTGATATGC-3’). We added an error-correcting 12-bp barcoded to the forward primer for multiplexing different samples and performed each PCR reaction in triplicate. PCR conditions were as follows: initial heating to 98^o^C for 30s, 30 cycles of denaturation at 98^o^C (10s), annealing at 53^o^C (20s) and extension at 72^o^C (10s), followed by a 7-min extension at 72^o^C before storage at 4^o^C; Conditions in the second PCR were identical to those in the first, with the exception that the denaturing-annealing-extension cycle was repeated only 10 times [33]. We pooled the PCR products in equimolar concentrations, purified by using E.Z.N.A.^®^ Gel Extraction Kit (Omega BioTek, Doraville, USA) and sequenced on a 2×250 bp paired-end Illumina MiSeq platform.

**Sequence processing**

We removed the short and low-quality sequences from raw sequencing data and merged the paired-end reads together following the pipelines of Mothur [49]. After removing the chimeric sequences by UCHIME [50], the high-quality sequences were clustered into operational taxonomic units (OTUs) at 97% sequences similarity using USEARCH [51]. Taxonomic classification of each OTU was determined using the RDP classifier with a confidence interval of 80% trained on the UNITE database [52]. Singletons and non-fungal sequences were excluded for subsequent analyses. Finally, to keep a consistent sequencing depth, we resampled 3,000 high-quality sequences for each root tip sample and 4,100 high-quality sequences for each background soil sample.

**Core root-associated fungal communities**

The profiles of root-associated fungi can be influenced by the surrounding soil environment [1, 3, 15], such as background soil properties and fungal communities. Thus, the root fungal associations for a given plant species may vary among individuals, especially across markedly heterogeneous soil environments. To obtain the representative root-associated fungal communities for each plant species, we defined core root-associated fungal OTUs according to their detected frequency (i.e., cutoffs) across all the root tip samples of a given plant species. For example, core OTUs at cutoff = 0.5 indicated that these OTUs could be found in half of the root tip samples of a given plant species. In this study, we defined core OTUs at nine different cutoffs ranging from 0.5 to 0.9 with intervals of 0.05. We conducted the subsequent analyses using the datasets of the overall OTUs and core OTUs to examine the consistency of the results.

**Phylogenetic relatedness of plant species**

In our previous study, we constructed the molecular phylogeny for the selected plant species using the sequences of four genes (i.e., *rbcL*, *matK*, *ITS1*, and *5.8S*) that were obtained from GenBank [29]. Briefly, two early-diverging gymnosperm species, *Abies alba* and *Cycas rumphii*, were added as the outgroup. After the alignment of sequences using ClustalX2 [60] and the determination of the best-fit nucleotide substitution model using Smart Model Selection (SMS) [61, 62], a maximum likelihood phylogenetic tree was constructed using PHYML 3.0 with a BIONJ starting tree [60]. The phylogeny was rooted by Archaeopteryx [63], and problematic sequences that were identified by BLAST were eliminated. We finally removed the two species in the outgroup to obtain a final molecular phylogeny (Fig. 1) and calculated the pairwise phylogenetic distances of plant species based on this maximum likelihood phylogenetic tree using the ‘ape’ package (*cophenetic.phylo* function) in R (Table S2).

**Functional traits of plant species**

The functional traits of the 39 plant species in this study were downloaded from the TRY website (<https://www.try-db.org/)> [32]. Specifically, we explored the records of functional traits based on the species names of our target plants. Traits with records for more than 13 target plant species were requested, downloaded and summarized (Table S3). A total of 11 functional traits were obtained from the TRY database, including growth form, plant woodiness, leaf phenology, leaf type, wood density, plant height, seed mass, plant tolerance to frost, leaf N, leaf P, and leaf N/P. A total of 21 plant species with more than 5 records of these functional traits were used for the calculation of functional distance (Gower dissimilarity) using the ‘FD’ package (*gowdis* function) in R (Table S2).

**Spatial co-occurrence patterns of plant species**

The spatial density and structure of individuals are essential to infer the role of local interactions on co-occurrence patterns between tree species. Forest inventory data provides this necessary information [13, 40, 64, 65]. In this study, we extracted the detailed spatial location of every individual of the 39 plant species in the 50-ha forest plot from the forest inventory data. We estimated the ratio of the observed mean density of a neighboring plant species (species *j*) at a given distance *r* from a focal plant species (species *i*) to the average density of this neighbor (species *j*) in the whole study region by applying bivariate pair correlation function *g_ij_*(*r*) [21-23]. Specifically, for a stationary Poisson process (i.e., completely random, spatial average densities of individuals for a given plant pair are independent), the pair correlation function *g_ij_*(*r*) is identically equal to 1 for all values of *r*. Values of *g_ij_*(*r*) > 1 indicate aggregation, suggesting that there are more individuals of plant species *j* than the spatial average at a distance *r* away from plant species *i*. Conversely, values of *g_ij_*(*r*) < 1 indicate segregation between this pair of plants at a distance *r*. The results of pair correlation function *g_ij_*(*r*) describe the spatial relationship of *i* and *j* as a function of the distance *r*, which help us to make inferences about their scale-dependent co-occurrence patterns and improve our ability in discriminating the effects of different ecological processes on plant co-occurrence [59-62]. We calculated the *g_ij_*(*r*) values of all pairs of the 39 plant species using the ‘spatstat’ package (*pcfcross* functions) in R. We estimated the spatial relationships (i.e., aggregation or segregation) for all the 741 pairs of plant species by extracting the values of *g_ij_*(*r*) at *r* = 1 m, where it is expected that root-associated microbial communities have a much larger impact on plant-plant interactions (Table S4).

**Spatial variation of soil environmental properties**

For the background soil environmental properties, pH and electrical conductivity (EC) were measured by specific electrodes. Soil moisture content was calculated based on the sample weight before and after air drying. Total carbon (TC) was measured by Shimadzu TOC Analyzer (TOC-V_CSH_; Shimadzu, Kyoto, Japan). Total nitrogen (TN), ammonium (NH_4_^+^) and nitrate (NO_3_^-^) were determined by SmartChem discrete autoanalyzer (SmartChem^®^200; WESTCO Scientific Instruments Inc.). Available P, Ca, Mg, Al, and K was exacted using Mehlich-III extractant solution and measured by inductively coupled plasma optical emission spectrometry (ICP-OES; Optima 2100DV; Perkin-Elmer, Massachusetts, USA). All the spatial location and environmental properties of background soil samples are provided in Table S5.

Based on the measured environmental properties, we estimated the soil environmental properties for the whole 50-ha plot in 10 × 10 m grid cells by applying geostatistical interpolation technique of ordinary kriging (*krige* function in ‘gstat’ package in R). Subsequently, we performed Principal Component Analysis (PCA) based on these interpolated environmental data and mapped the PC1 values to show the variation of soil environmental properties [70] in this 50-ha subtropical forest dynamic plot (Fig. S3b). The associated soil environmental properties of each plant individuals from which we collected root tip samples were estimated according to their spatial locations (Table S6).

**REFERENCES**

1. Bever JD, Mangan SA, Alexander HM. Maintenance of plant species diversity by pathogens. Annu Rev Ecol Evol Syst. 2015;46:305-25.

3. Tedersoo L, Bahram M, Zobel M. How mycorrhizal associations drive plant population and community biology. Science. 2020;367:eaba1223.

13. Eppinga MB, Baudena M, Johnson DJ, Jiang J, Mack KM, Strand AE, et al. Frequency-dependent feedback constrains plant community coexistence. Nat Ecol Evol. 2018;2:1403-7.

15. van der Linde S, Suz LM, Orme CDL, Cox F, Andreae H, Asi E, et al. Environment and host as large-scale controls of ectomycorrhizal fungi. Nature. 2018;558:243-8.

21. Wiegand T, Moloney KA. Rings, circles, and null‐models for point pattern analysis in ecology. Oikos. 2004;104:209-29.

22. Perry GL, Miller BP, Enright NJ. A comparison of methods for the statistical analysis of spatial point patterns in plant ecology. Plant Ecol. 2006;187:59-82.

23. Law R, Illian J, Burslem DF, Gratzer G, Gunatilleke CV, Gunatilleke IA. Ecological information from spatial patterns of plants: insights from point process theory. J Ecol. 2009;97:616-28.

29. Chen Y, Jia P, Cadotte MW, Wang P, Liu X, Qi Y, et al. Rare and phylogenetically distinct plant species exhibit less diverse root-associated pathogen communities. J Ecol. 2019;107:1226-37.

30. Peters HA. Neighbour‐regulated mortality: the influence of positive and negative density dependence on tree populations in species‐rich tropical forests. Ecol Lett. 2003;6:757-65.

32. Kattge J, Diaz S, Lavorel S, Prentice IC, Leadley P, Bönisch G, et al. TRY - a global database of plant traits. Glob Chang Biol. 2011;17:2905-35.

33. Davey ML, Heegaard E, Halvorsen R, Ohlson M, Kauserud H. Seasonal trends in the biomass and structure of bryophyte-associated fungal communities explored by 454 pyrosequencing. New Phytol. 2012;195:844-56.

40. LaManna JA, Belote RT, Burkle LA, Catano CP, Myers JA. Negative density dependence mediates biodiversity-productivity relationships across scales. Nat Ecol Evol. 2017;1:1107-15.

55. Toju H, Sato H, Yamamoto S, Kadowaki K, Tanabe AS, Yazawa S, et al. How are plant and fungal communities linked to each other in belowground ecosystems? A massively parallel pyrosequencing analysis of the association specificity of root‐associated fungi and their host plants. Ecol Evol. 2013;3:3112-24.

56. Schloss PD, Westcott SL, Ryabin T, Hall JR, Hartmann M, Hollister EB, et al. Introducing mothur: Open-source, platform-independent, community-supported software for describing and comparing microbial communities. Appl Environ Microbiol. 2009;75: 7537-41.

57. Edgar RC, Haas BJ, Clemente JC, Quince C, Knight R. UCHIME improves sensitivity and speed of chimera detection. Bioinformatics. 2011;27:2194-200.

58. Edgar RC. Search and clustering orders of magnitude faster than BLAST. Bioinformatics. 2010;26:2460-1.

59. Abarenkov K, Henrik Nilsson R, Larsson KH, Alexander IJ, Eberhardt U, Erland S, et al. The UNITE database for molecular identification of fungi - recent updates and future perspectives. New Phytol. 2010;186:281-5.

60. Larkin MA, Blackshields G, Brown NP, Chenna R, McGettigan PA, McWilliam H, et al. Clustal W and Clustal X version 2.0. Bioinformatics. 2007;23:2947-8.

61. Guindon S, Gascuel O. A simple, fast, and accurate algorithm to estimate large phylogenies by maximum likelihood. Syst Biol. 2003;52:696-704.

62. Anisimova M, Gascuel O. Approximate likelihood-ratio test for branches: a fast, accurate, and powerful alternative. Syst Biol. 2006;55:539-52.

63. Zmasek CM, Eddy SR. ATV: display and manipulation of annotated phylogenetic trees. Bioinformatics. 2001;17:383-4.

64. Johnson DJ, Beaulieu WT, Bever JD, Clay K. Conspecific negative density dependence and forest diversity. Science. 2012;336:904-7.

65. LaManna JA, Mangan SA, Alonso A, Bourg NA, Brockelman WY, Bunyavejchewin S, et al. Plant diversity increases with the strength of negative density dependence at the global scale. Science. 2017;356:1389-92.

66. Getzin S, Wiegand T, Wiegand K, He F. Heterogeneity influences spatial patterns and demographics in forest stands. J Ecol. 2008;96:807-20.

67. Brown C, Law R, Illian JB, Burslem DF. Linking ecological processes with spatial and non-spatial patterns in plant communities. J Ecol. 2011;99:1402-14.

68. Brown C, Burslem DF, Illian JB, Bao L, Brockelman W, Cao M, et al. Multispecies coexistence of trees in tropical forests: spatial signals of topographic niche differentiation increase with environmental heterogeneity. Proc R Soc B Biol Sci. 2013;280:20130502.

69. Benot ML, Bittebiere AK, Ernoult A, Clément B, Mony C. Fine-scale spatial patterns in grassland communities depend on species clonal dispersal ability and interactions with neighbours. J Ecol. 2013;101:626-36.

70. John R, Dalling JW, Harms KE, Yavitt JB, Stallard RF, Mirabello M, et al. Soil nutrients influence spatial distributions of tropical tree species. Proc Natl Acad Sci USA. 2007;104:864-9.
