# Supplementary material for: Root-associated fungal community reflects host spatial co-occurrence patterns in a subtropical forest
Source: ISME Commun. 2021 Nov 6;1:65. doi: 10.1038/s43705-021-00072-6 (PMC9723750; doi:10.1038/s43705-021-00072-6)
Supplement: Supplementary file 2 — Supplementary Figures [file 43705_2021_72_MOESM2_ESM.pdf]

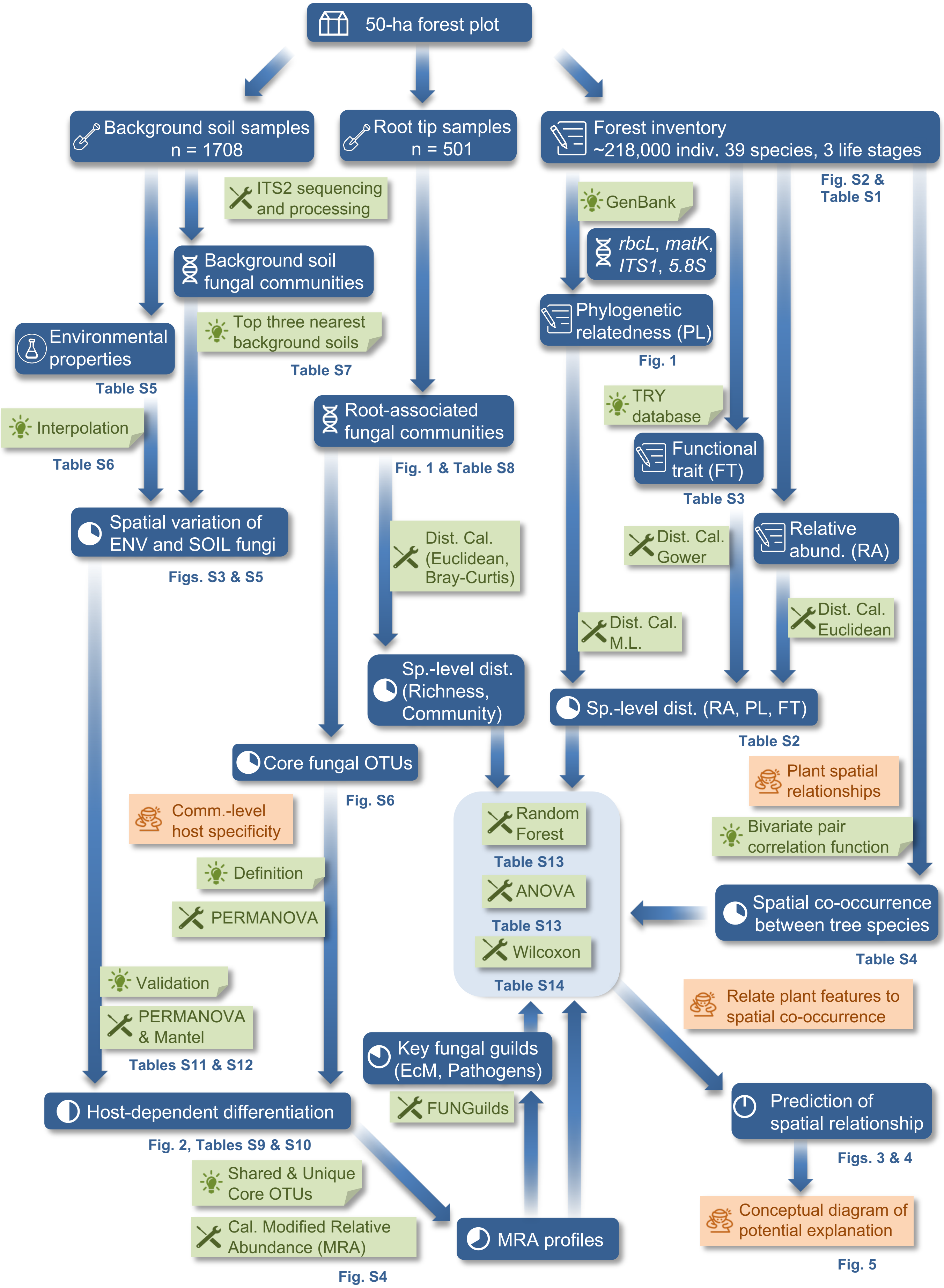

Fig. S1 The detailed workflow and key description of the materials and methods in this study.

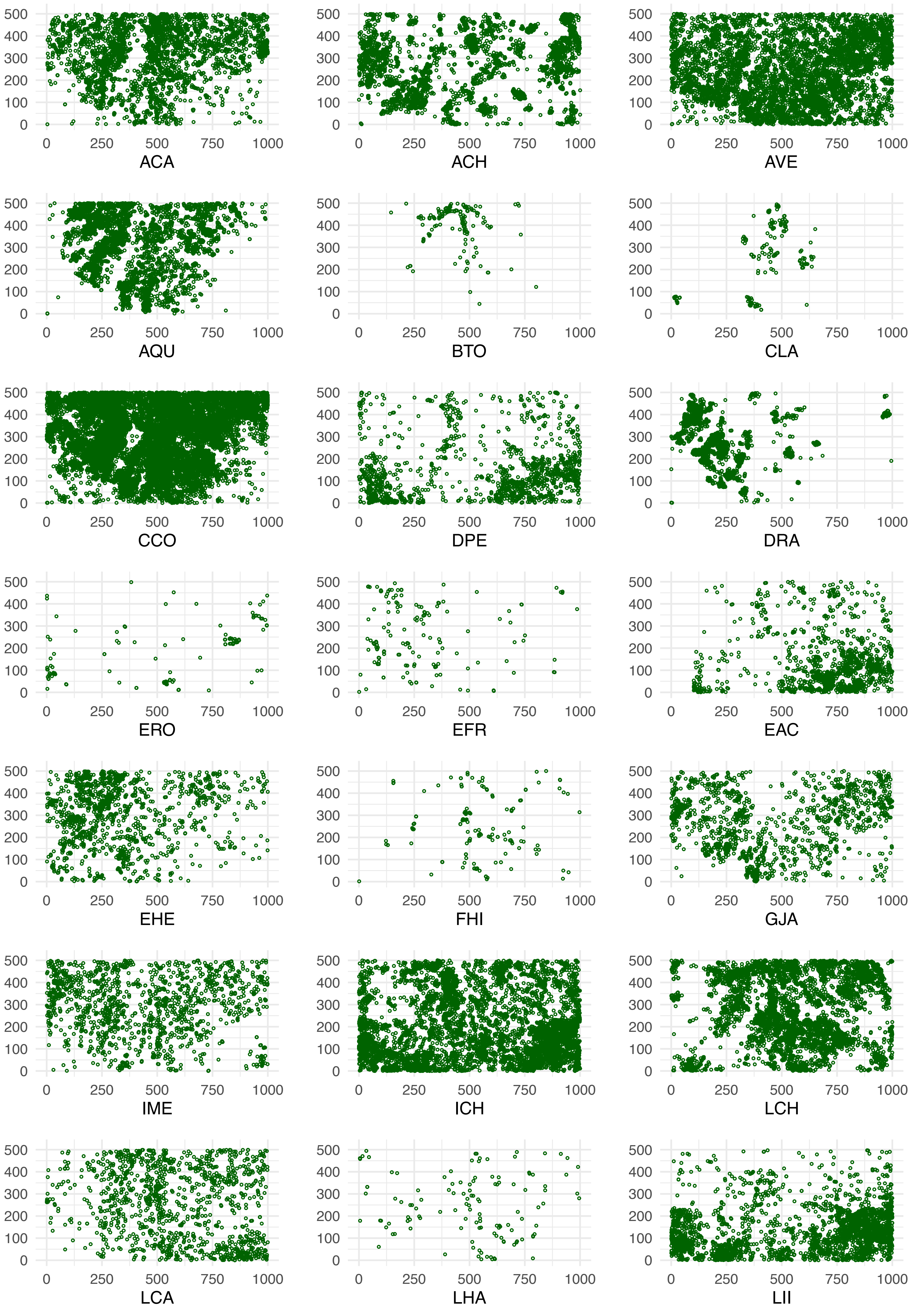
ACA
ACH
AVE
AQU
BTO
CLA
CCO
DPE
DRA
ERO
EFR
EAC
EHE
FHI
GJA
IME
ICH
LCH
LCA
LHA
LII

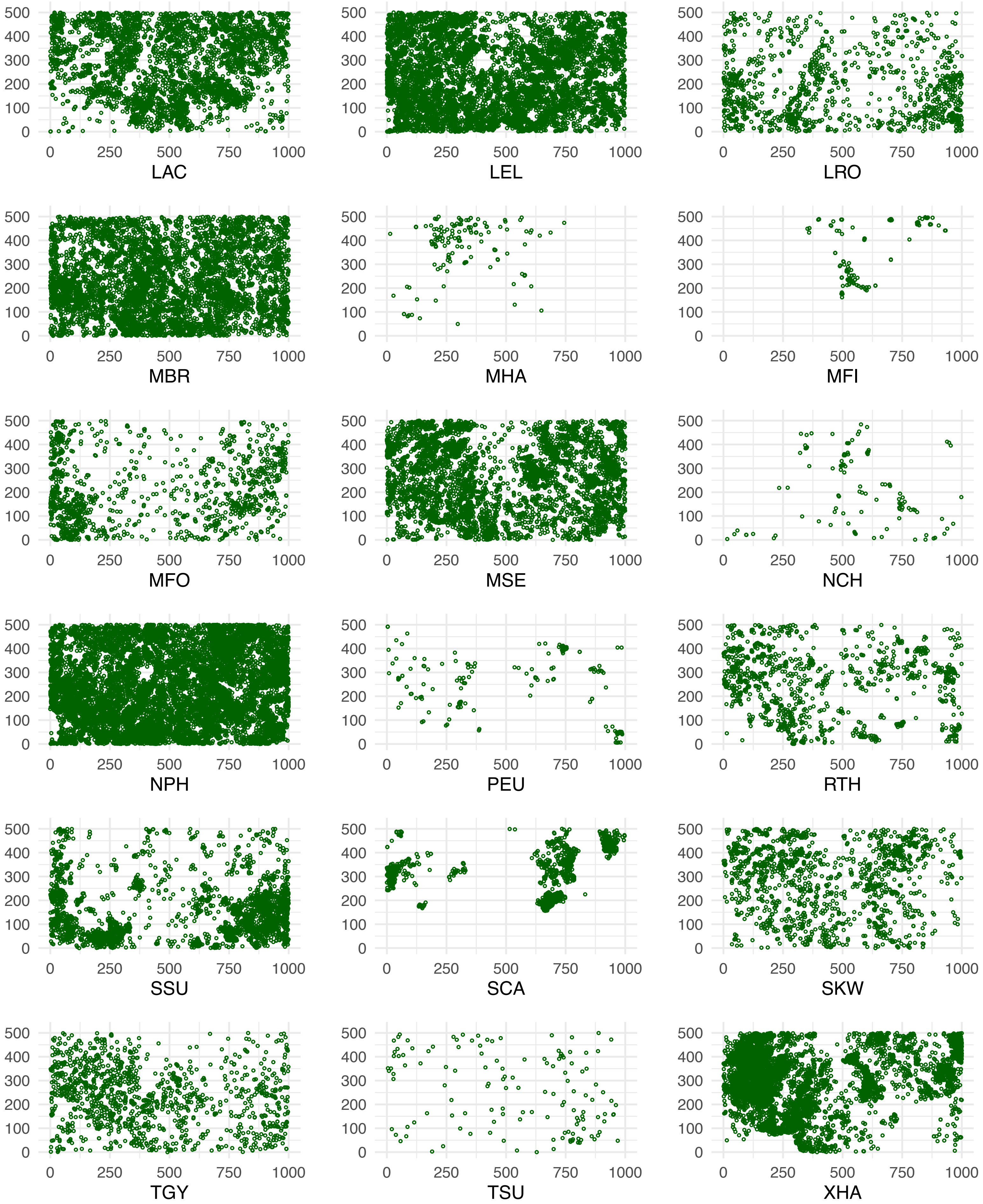

Fig. S2 Spatial distribution of the 39 plant species in the 50-ha forest plot.

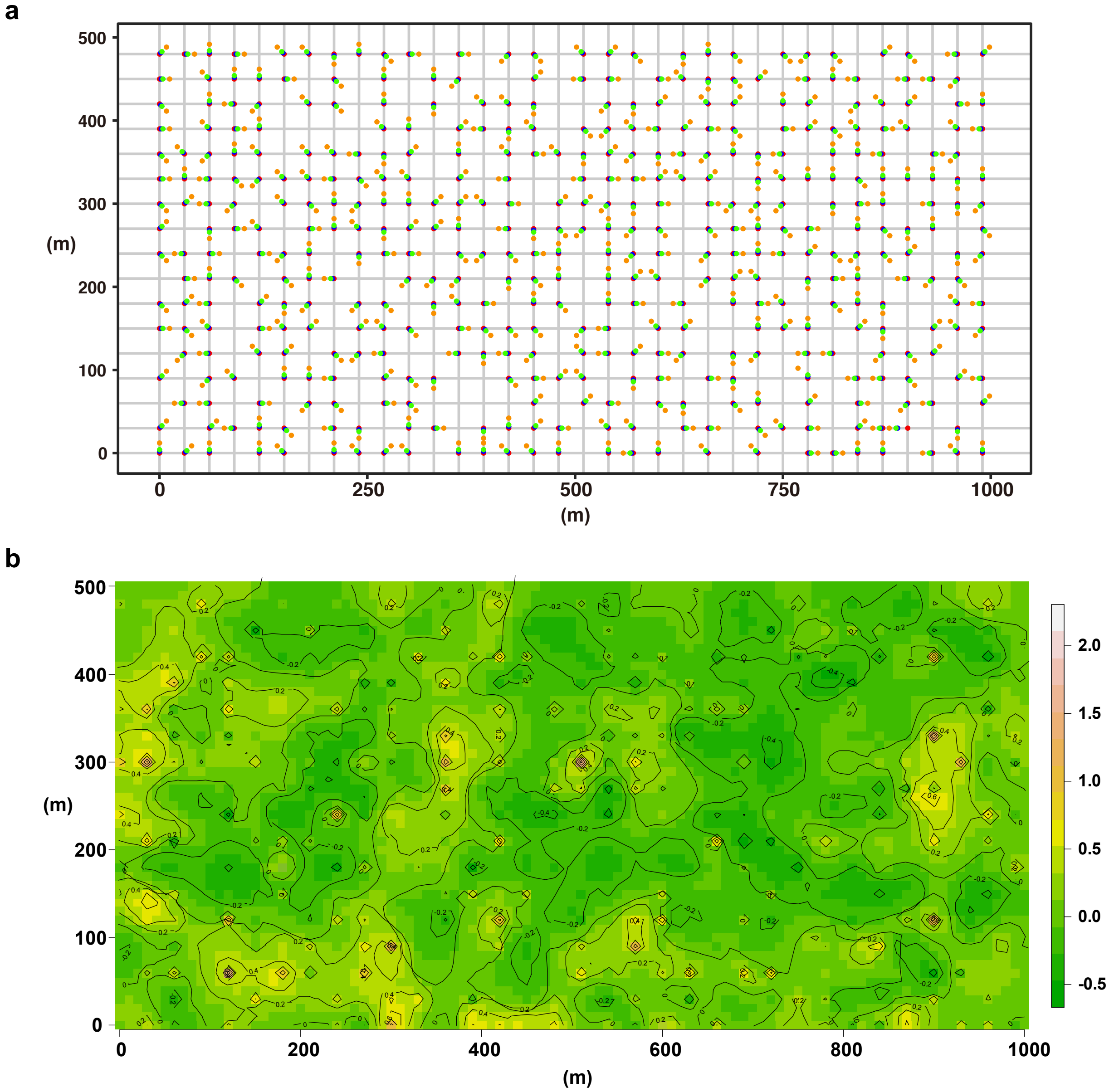

Fig. S3 Spatial distribution of background soil samples and the contour map of PC1 values of environmental properties in the 50 ha forest plot. a, Red dots are the 427 grid points of the 30 x 30 m grid cells. Blue, green and orange dots are sampling points which are 2 m, 4 m, 12 m away from the grid points, respectively. **b**, Soil environmental properties in 10 x 10 m grid cells were estimated using ordinary kriging based on the measured values of background soils (Table S5). Principal Component Analysis (PCA) were performed based on these interpolated environmental data and the PC1 values were mapped to show the variation of soil environmental properties.

Relative abundances (RA) and modified relative abundances (MRA) of core fungal OTUs

Core fungal OTUs	Sp.1	Sp.2	Shared core OTUs		Unique core OTUs	
			Product of RA	MRA	RA	MRA
OTU1	0.35	0.15	0.0525	0.6325	0	0
OTU2	0.15	0.15	0.0225	0.2711	0	0
OTU3	0.2	0.01	0.002	0.0241	0	0
OTU4	0.1	0.02	0.002	0.0241	0	0
OTU5	0	0	0	0	0	0
OTU6	0.05	0	0	0	0.05	0.1389
OTU7	0	0.01	0	0	0.01	0.0278
OTU8	0.02	0	0	0	0.02	0.0556
OTU9	0	0.02	0	0	0.02	0.5556
OTU10	0.05	0	0	0	0.05	0.1389
OTU11	0.01	0.35	0.0035	0.0422	0	0
OTU12	0.05	0.01	0.0005	0.006	0	0
OTU13	0.01	0	0	0	0.01	0.0278
OTU14	0	0.1	0	0	0.1	0.0278
OTU15	0.01	0	0	0	0.01	0.0278
**Subtotal**	**1**	**1**	**0.083**	**1**	**0.36**	**1**

Profiles of MRA for shared and unique OTUs

Shared MRA	OTU1	OTU2	OTU3	OTU4	OTU5	OTU6	OTU7	OTU8	OTU9	OTU10	OTU11	OTU12	OTU13	OTU14	OTU15
Sp.1 ~ Sp.2	0.6325	0.2711	0.0241	0.0241	0	0	0	0	0	0	0.0422	0.006	0	0	0

Unique MRA	OTU1	OTU2	OTU3	OTU4	OTU5	OTU6	OTU7	OTU8	OTU9	OTU10	OTU11	OTU12	OTU13	OTU14	OTU15
Sp.1 ~ Sp.2	0	0	0	0	0	0.1389	0.0278	0.0556	0.5556	0.1389	0	0	0.0278	0.0278	0.0278

Fig. S4 An example showing the calculation procedure of the modified relative abundance (MRA) of different fungal species using their relative abundance (RA) data. For each plant pair, we split the root-associated fungal species into shared (i.e., detected in both plants) and unique (i.e., detected in either plant) core OTUs to differentiate the influences from potential, pairwise 'generalists' and 'specialists', respectively. We calculated the modified relative abundance to evaluate the relative abundance of each shared (or unique) core fungal OTU. Specifically, for the MRA of unique core OTUs, we divided their relative abundances by the sum of relative abundances from all unique core OTUs. For the MRA of shared core OTUs, we divided their products of relative abundances by the sum of each product between the two relative abundances of shared core OTUs. Thus, for a pair of plants, the sum of MRA of all shared (or unique) core fungal OTUs was adjusted to 1. A fungal OTU had a higher value of MRA implied higher recruitment of this fungal species when plants grew together. We applied this calculation procedure for all pairs of different plant species and the pairs of plant species at their different life stages. In addition, we calculated these profiles of modified relative abundances based on the core OTUs at cutoffs of 0.5 and 0.9. We obtained the MRA profiles for shared (or unique) OTUs and used them as the input predictor variables in Random Forest modeling.

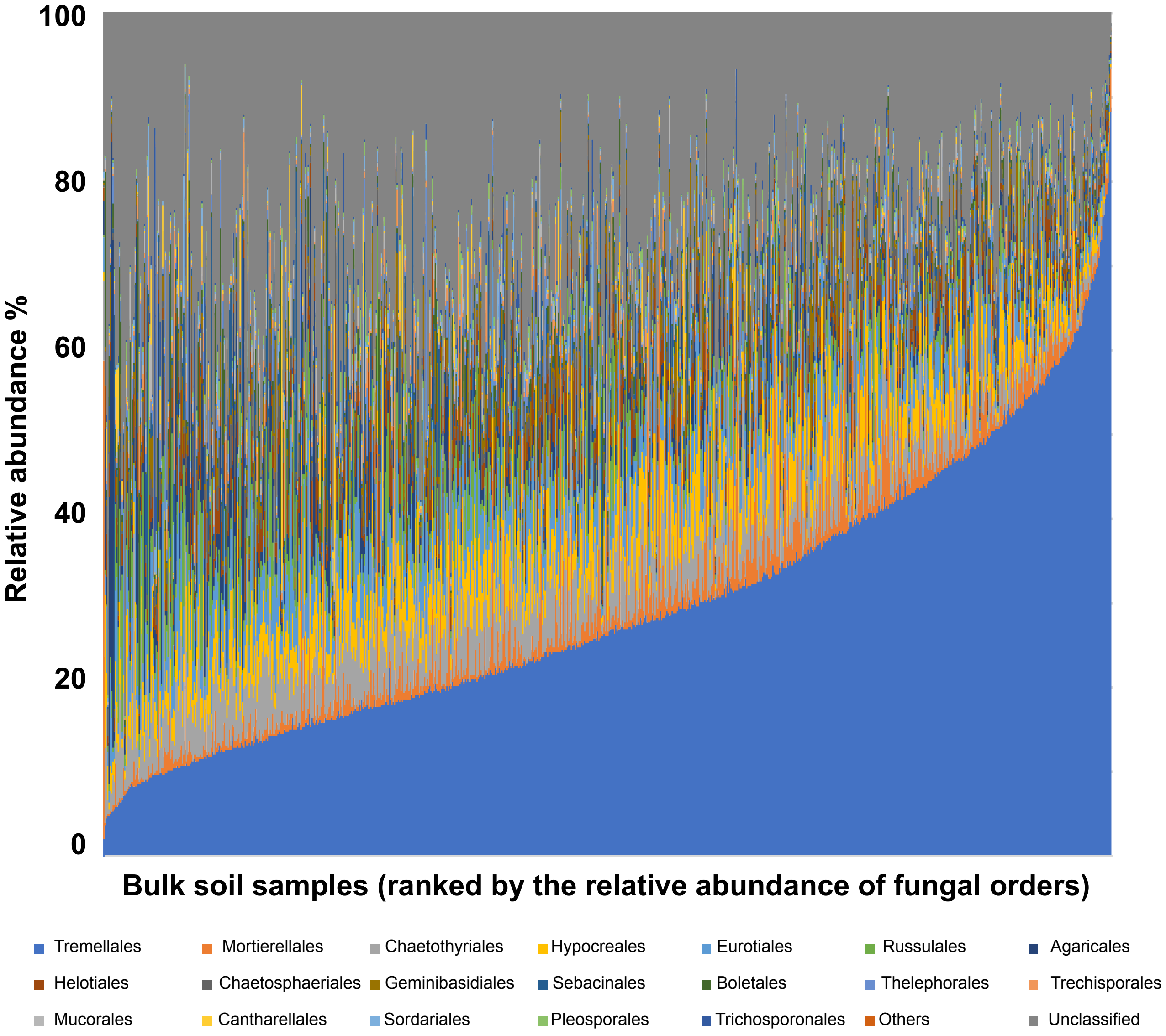

Fig. S5 Fungal community composition of all 1708 bulk soil samples at order level.

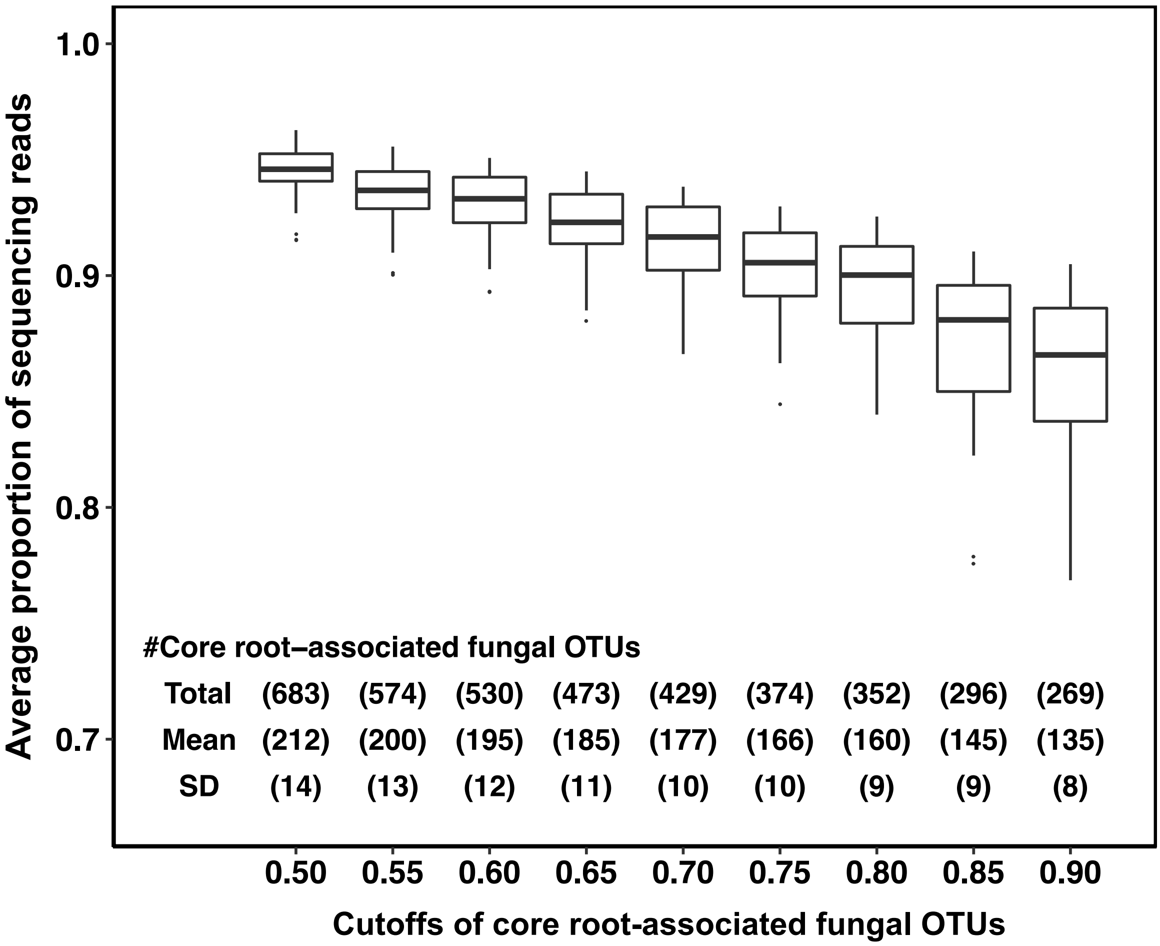

Fig. S6 Species richness of core root-associated fungi among different cutoffs and the average proportion of sequencing reads (mean ± s.d.) that were represented by these core OTUs. For a given plant species, the cutoffs of core OTUs indicated their detected frequency across all the root tip individuals. For example, core OTUs at cutoff = 0.5 indicated that these OTUs could be found in half of the root tip samples of a given plant species.
